# High fidelity heralded single-photon source using cavity quantum electrodynamics

**DOI:** 10.1038/s41598-018-21481-z

**Published:** 2018-02-16

**Authors:** Xin Zhang, Chang Xu, Zhongzhou Ren

**Affiliations:** 1 Department of Physics, Nanjing University, Nanjing, 210008 China; 2 School of Physics Science and Engineering, Tongji University, Shanghai, 200092 China

## Abstract

Demands for single-photon sources are ubiquitous in quantum information processing as well as in quantum metrology. In many protocols for producing single photons, a cavity-emitter configuration is used. In such cavity quantum electrodynamical systems, the cavity can enforce a well-defined output mode for the photon and enhance its collection efficiency, while the emitter is indispensable for single photon emission. Here we show the two cavity-one two-level emitter configuration can be used to produce exclusively photon pairs, with each photon in a separate mode. Conditioning on detecting a photon in one of the modes, one heralds with high fidelity a single photon in the other mode. Counterintuitively, upon decreasing the coupling of the emitter to one of the modes, the heralding fidelity can further increase.

## Introduction

Single photons are very important for various quantum information processing tasks as well as in quantum metrology applications^1,2^. For example, using single photons as the computing resource, linear quantum computing^3,4^, and algorithms like boson sampling^5^ can carry out tasks intractable for classical computers. Also, single photons can drastically extend the reach of quantum repeaters and greatly facilitate device independent quantum key distribution (DI-QKD) tasks^6^. Single photons can benefit metrology as well. For example, absorption measurements using single photons has no shot noise and thus the smallest amount of absorption measurable is not shot-noise limited^7^. Single photons can also be used, either by themselves^8^ or by building up multiphoton states, to resolve smaller features or attain smaller statistical error than classical light^7,9^. In many protocols for producing single photons, a cavity-emitter configuration is used^10–24^. The cavity can enforce a single output mode for the produced photon and enhance its collection efficiency, while the emitter is indispensable for single photon emission. In this paper we propose a protocol to produce with high fidelity single photon states using the two-mode Jaynes-Cummings^25^ model, namely a two cavity mode-one two-level emitter configuration. This protocol works by producing exclusively photon pairs, with one photon in each mode. Conditioning on detecting a photon in one mode, one can know (herald) with high fidelity that the other mode is in a single photon state.

## Results

### Concepts

**Figure 1:**
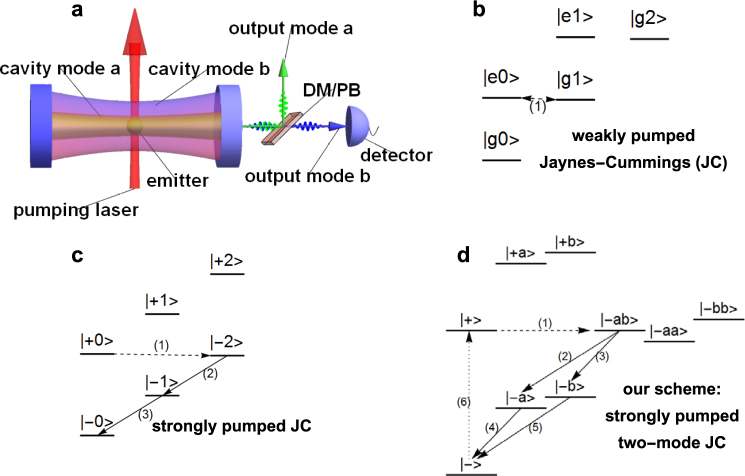
Concepts of the proposal. (**a)** Schematic of the proposed protocol. This protocol can produce exclusively photon pairs with each photon in a separate mode. Since the two modes are designed to have different energy, and probably different polarization, the two photons can be separated with a dichroic mirror or a polarization beam splitter (DM/PB in the plot). Conditioning on detecting a photon in one mode, for example in mode *b* as depicted here, one can herald with high fidelity a single photon state in the other mode *a*. (**b**–**d**) The comparison and “evolution” from the energy level diagram of the typical weakly pumped Jaynes-Cummings model to our scheme. (**b)** The typical energy level diagram for the weakly pumped Jaynes-Cummings model. (**c**) Strongly pumped Jaynes Cummings model. (**d)** Our scheme, strongly pumped two-mode Jaynes-Cummings model. For more details please see the text.

First we explain the concepts of the proposed protocol. The proposed experimental setup is depicted in Fig. 1a. A two-level emitter is positioned inside a two-mode cavity. The system is assumed to be in the strong coupling regime such that the coherent energy exchange between the cavity modes and the emitter is considerably faster than both the cavity decay rates and the spontaneous emission rate of the emitter. A strong driving laser comes in from the side and shines on the emitter.

The system in our protocol (cf. Figure 1a) obeys the master equation^26,27^:

1 $$\begin{array}{rcl}\frac{d\rho }{dt} & = & -i[H,\rho ]+{\kappa }_{a}[a\rho {a}^{\dagger }-\frac{1}{2}{a}^{\dagger }a\rho -\frac{1}{2}\rho {a}^{\dagger }a]\\  &  & +{\kappa }_{b}[b\rho {b}^{\dagger }-\frac{1}{2}{b}^{\dagger }b\rho -\frac{1}{2}\rho {b}^{\dagger }b]\\  &  & +{\gamma }_{\sigma }[{\sigma }_{-}\rho {\sigma }_{+}-\frac{1}{2}{\sigma }_{+}{\sigma }_{-}\rho -\frac{1}{2}\rho {\sigma }_{+}{\sigma }_{-}],\end{array}$$

where *ρ* is the density matrix of the system and *H* is the Hamiltonian given by (written in the rotating frame of the laser):

2 $$\begin{array}{rcl}H & = & {\delta }_{\sigma }{\sigma }_{+}{\sigma }_{-}+{\rm{\Omega }}({\sigma }_{+}+{\sigma }_{-})+{\delta }_{a}{a}^{\dagger }a+{\delta }_{b}{b}^{\dagger }b\\  &  & +{g}_{a}(a{\sigma }_{+}+{a}^{\dagger }{\sigma }_{-})+{g}_{b}(b{\sigma }_{+}+{b}^{\dagger }{\sigma }_{-}).\end{array}$$

Here *σ*_−_ = |*g*〉 〈*e* | where |*g*〉 and |*e*〉 are respectively the ground and excited states of the two-level emitter. *σ*_+_ = (*σ*_−_)^†^, while *a*, *b* are the annihilation operators for the cavity modes *a* and *b*. *δ*_*σ*_, *δ*_*a*_, *δ*_*b*_ are respectively the detunings of the emitter, and those of the cavity mode *a* and the cavity mode *b* with respect to the driving laser, respectively. Ω is the pumping strength of the laser. *g*_*a*_, *g*_*b*_ are the coupling constants between the emitter and the cavity modes *a* and *b* respectively. *κ*_*a*_, *κ*_*b*_ and *γ*_*σ*_ are the decay rates of the cavity mode *a*, that of the cavity mode *b*, and the spontaneous emission rate of the two-level emitter respectively.

The |+〉, |*−*〉 states are the eigenstates of the part of *H* that corresponds to the laser-pumped emitter, namely, the laser dressed states:

3 $$[{\delta }_{\sigma }{\sigma }_{+}{\sigma }_{-}+{\rm{\Omega }}({\sigma }_{+}+{\sigma }_{-})]|\pm \rangle ={E}_{\pm }|\pm \rangle .$$

To explain how our scheme works, in Fig. 1b–d we show the comparison and “evolution” from the weakly pumped Jaynes-Cummings model, to the strongly pumped Jaynes-Cummings model, and finally to our scheme. In Fig. 1b, the typical energy level diagram for the weakly pumped Jaynes-Cummings model is shown. Here the states can be labeled by specifying whether the two-level emitter is in the ground (g) or the excited (e) state, and the photon number of the cavity. When the system states |*e*0〉 and |*g*1〉 is tuned into resonance, population is transferred coherently between them (dashed double edged arrow labeled “(1)” in Fig. 1b).

In Fig. 1c, a strong pumping is added. Now the two-level system is dressed by the pumping laser. Namely, the ground and excited states are no longer eigenstates but are strongly mixed with each other. Now the states are labeled by which dressed state (|+〉 or |−〉) (cf. equation (3)) the two-level system is in, and the cavity photon number. If, for example |+0〉 and |−2〉 is tuned into resonance, the population in |+0〉 will be transfered coherently to |−2〉 (dashed arrow (1) in Fig. 1c). Subsequently, via the cavity decay route |−2〉 → |−1〉 → |−0〉 a photon pair will be generated (solid arrows (2) and (3) in Fig. 1c)^28^.

In Fig. 1d, another cavity mode is added. This is the energy-level diagram of the present work. The states can be labeled by specifying the dressed state the emitter is in, and the photon occupation in each cavity modes. The system state |+〉 denotes the emitter in the dressed state |+〉 and no photon in either cavity, |+*a*〉 denotes the emitter in state |+〉 and one photon in mode *a*, while |−*ab*〉 denotes the emitter in |−〉 with one photon in each mode, etc. By tuning carefully the frequency of the driving laser, the state |−*ab*〉 is tuned into resonance with the state |+〉. The system for most of the time resides in the zero-photon manifold consisting of the system states |+〉 and |−〉. The photon pair generation works as follows: Starting from the state |+〉, the system is coherently transferred to the state |−*ab*〉 (the dashed arrow labeled “(1)” in Fig. 1d). Transfers to states with a single photon such as |−*a*〉 or |+*b*〉 are highly suppressed due to large detuning. The two cavity photons then decay out of the cavity and the system ends up in the state |−〉 (the solid arrows (2) and (4), as well as (3) and (5), in Fig. 1d). Finally a spontaneous emission of the emitter (dotted arrow (6) in Fig. 1d) projects the emitter with some probability into the state |+〉 and the above process starts again.

In each cycle, since the two emitted cavity photons originate from different cavity modes, they have different energy and probably different polarization. Consequently they can be separated by a dichroic mirror or a polarizing beam splitter. Conditioning on detecting one photon in one mode, one can know with high confidence that the other mode is in a single photon state.

The design of the present protocol is guided by the principle of energy selection. By working at the two-photon resonance, two-photon emissions are favored, while single-photon emissions are largely suppressed because the occupation of single-photon states |±*a*〉 and |±*b*〉 are suppressed due to large detuning. Guided by the same principle, by employing two modes with different energies, the probability of two photon being in the same mode is highly suppressed, while the probability of two photon being in different modes is favored. These can enable exclusive emission of separable photon pairs, which in turn enables a near-unity heralding fidelity.

The coherent transfer between the states |+〉 and |−*ab*〉 can be understood as follows. Since the energies of these two states are tuned to be degenerate, any existent coupling between them can effectively transfer population from one to the other. The actual coupling between them is a second-order process^28^ mediated by the single photon states. The system Hamiltonian first couples the state |+〉 to the intermediate single photon states |± *a*〉 and |± *b*〉, and in a second step couples these states to |−*ab*〉. Due to the large detuning, the intermediate single-photon states are only virtually populated.

### Steady state analysis

**Figure 2:**
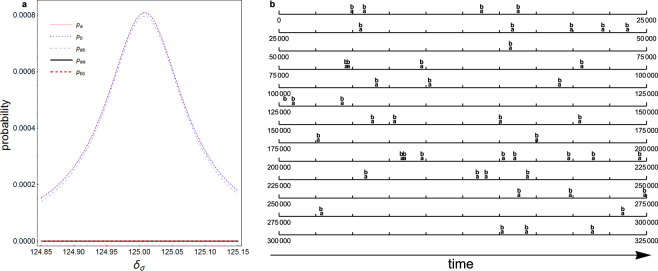
Steady state analysis and quantum trajectory simulation. (**a**) The steady-state probabilities of having one photon in mode *a* (*p*_*a*_), one photon in mode *b* (*p*_*b*_), and one photon in each mode (*p*_*ab*_), etc., are plotted against the laser detuning with respect to the two-level emitter. The states |+〉 and |−*ab*〉 are degenerate with each other at the center. The system for most of the time resides in the zero-photon manifold consisting of the system states |+〉 and |−〉. The corresponding probability *p*_0_ is not shown. (**b**) An exemplary record from the quantum trajectory simulation. Photons are essentially always emitted in (*ab*) pairs. The horizontal axes represent time. The times when a cavity emission in mode *a* or mode *b* occurs are marked by the letters (**a**) and (**b**), respectively. The parameters are Ω = 150, *δ*_*a*_ − *δ*_*σ*_ = 25, *δ*_*b*_ − *δ*_*σ*_ = 50, *g*_*a*_ = *g*_*b*_ = 1, *κ*_*a*_ = *κ*_*b*_ = 0.1, *γ*_*σ*_ = 0.01. The calculation methods are given in Methods. For more details please see the text.

A quantitative analysis of the protocol using the steady state solution of the master equation^26–29^ is given in Fig. 2a. As can be seen from the figure, when the state |+〉 is tuned into degeneracy with the state |−*ab*〉, the probability *p*_*ab*_ for having one photon in each mode reaches a maximum while the probabilities for having two photon in the same mode *p*_*aa*_ and *p*_*bb*_ remain negligibly small. Also, around the maximum point, one has approximately *p*_*ab*_ × *κ*_*b*_ = *p*_*a*_ × *κ*_*a*_, *p*_*ab*_ × *κ*_*a*_ = *p*_*b*_ × *κ*_*b*_, where *p*_*a*_, *p*_*b*_ are respectively the probability for having one photon in mode *a* and that for having one photon in mode *b*. This occurs because essentially all the population of single photon states comes from the cavity decay of the state |−*ab*〉 to either |−*a*〉 or |−*b*〉. So, for example, the first equation simply means the feeding rate to *p*_*a*_ from the above decay (*κ*_*b*_ × *p*_*ab*_) is equal to its depletion rate (*p*_*a*_ × *κ*_*a*_).

### Quantum trajectory simulation results

Based on the reassurance from the above steady-state analysis, we proceed to perform extensive quantum trajectory^26,30–32^ simulations. In Fig. 2b, we show an examplary record of photon emissions given by the simulation. As can be seen, in virtually all cases, emissions occur in pairs. Also, each pair essentially always consists of one photon from each mode. To assess quantitatively the fidelity for heralding a single photon state, we identify two photons as being emitted together if they are emitted within a preset time window from each other. Likewise, for three or more photons, if each emission occur within the preset time window from the last emission, we count all these photons as emitted together. Ideally, one photon in mode *a* and one photon in mode *b* are always emitted together and form a pair, while the intervals between pairs are much longer. In such cases, the heralding fidelity would be unity. An exemplary result of the analysis is given in Table 1. As can be seen, by far the dominate emission is photon pairs with one photon in each mode. Conditioning on the detection of a photon in mode *a*, the probability of successfully heralding a single photon state in the other mode is the probability of emitting *ab* pairs divided by the probability of emitting at least one photon in mode *a*. Using the notations in Table 1, this means (*N*_*ab*_ + *N*_*ba*_)/(*N*_*tot*_ − *N*_*b*_ − *N*_*bb*_ − …). This gives 99.0%.

**Table 1:** Analysis of the heralding fidelity.

Notation	Sequence	Counts
*N*_*a*_	*a*	924
*N*_*b*_	*b*	828
*N*_*ab*_	*a* → *b*	53940
*N*_*ba*_	*b* → *a*	52992
*N*_*abab*_	*a* → *b* → *a* → *b*	36
*N*_*abba*_	*a* → *b* → *b* → *a*	48
*N*_*baab*_	*b* → *a* → *a* → *b*	48
*N*_*baba*_	*b* → *a* → *b* → *a*	24
*N*_*tot*_	all	108840

The first column denotes the name of the sequence, and the second column gives its schematic representation. For example, *a* → *b* means events where a photon emission in mode *a* is followed closely by one in mode *b*, while the intervals to nearby emissions are larger than a preset time window. The last column gives the number of counts. The last row gives the total counts *N*_tot_. The parameters are Ω = 150, *δ*_*a*_ − *δ*_*σ*_ = 25, *δ*_*b*_ − *δ*_*σ*_ = 50, *g*_*a*_ = *g*_*b*_ = 1, *κ*_*a*_ = *κ*_*b*_ = *κ* = 0.1, *γ*_*σ*_ = 0.01. The time window for identifying two photons as emitted together are chosen to optimize the heralding fidelity, as it can be tuned experimentally. The heralding fidelity conditioning on detecting a photon in mode *a* is (*N*_*ab*_ + *N*_*ba*_)/(*N*_tot_ − *N*_*b*_ − *N*_*bb*_ − …) = 99.0%. For more details please see the text.

**Figure 3:**
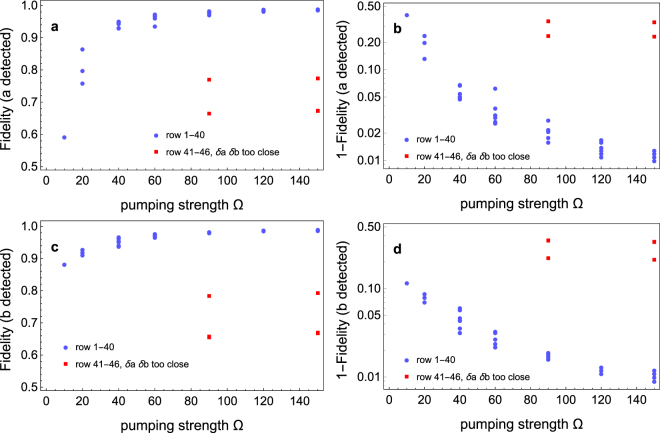
The heralding fidelity as a function of the laser pumping strength. The system parameters and detailed numerical data corresponding to each point are given in Table 2. (**a**) The heralding fidelity conditioning on detecting a photon in mode *a* as a function of the pumping strength Ω. The numerical data corresponding to the blue solid circles are given in row number 1–40 of Table 2. The red solid squares are cases where the resonant frequencies of the two cavities are chosen very close together. The corresponding numerical data are given in row number 41–46 of Table 2. (**b**) Same as in a, but here (1-fidelity) is shown instead of the fidelity itself to better resolve the changes close to unity, and a logarithmic scale is used. In this figure a lower point means higher heralding fidelity. (**c**,**d**), same as **a** and **b** respectively, but conditioning on detecting a photon in mode *b*. As can be seen, as a general trend the heralding fidelity increases with the pumping strength. For more details please see the text.

We perform extensive simulations for different parameter combinations. The results are given in Fig. 3 and Tables 2–4. As can be seen from the solid blue circles in Fig. 3, as a general trend larger pumping strength leads to higher heralding fidelity. In choosing experimental parameters, the two cavity modes should better be detuned from each other. Otherwise, states |−*aa*〉 and |−*bb*〉 would be degenerate with |−*ab*〉 and get populated considerably, giving lower heralding fidelity, as seen from the solid red squares in Fig. 3. The detailed parameters and numerical data corresponding to each point in Fig. 3 are given in Table 2. As can be seen by comparing the fifth and sixth columns of Table 2, the heralding fidelities conditioning on detecting a photon in different modes are generally different. This means one can choose to put the detector in the mode which gives higher heralding fidelity. While in general stronger pumping leads to higher fidelity as already seen in Fig. 3, weaker pumping in general leads to higher heralding rate, as seen from the last column of Table 2.

**Table 2:** Dependence of the heralding fidelity on the pumping strength and relative detunings.

Row no.	Ω	(*δ*_*a*_ − *δ*_*σ*_)	(*δ*_*b*_ − *δ*_*σ*_)	Fidelity (*a* detected)	Fidelity (*b* detected)	Rate
1	10	−25	50	59.3%	88.3%	0.00045
2	20	−25	50	86.6%	92.9%	0.0010
3	20	−20	5	76.0%	92.0%	0.00027
4	20	−10	10	79.9%	91.2%	0.00053
5	40	−20	5	94.5%	96.8%	0.00030
6	40	−10	10	95.2%	96.4%	0.00043
7	40	10	20	95.1%	95.6%	0.00081
8	40	20	25	94.9%	95.3%	0.0010
9	40	25	50	93.1%	93.9%	0.0016
10	40	30	60	93.2%	94.2%	0.0019
11	60	−25	50	93.7%	96.7%	0.00061
12	60	−20	5	97.0%	97.8%	0.00024
13	60	−10	10	97.3%	97.8%	0.00031
14	60	10	20	97.4%	97.6%	0.00050
15	60	20	25	97.3%	97.3%	0.00063
16	60	25	50	96.8%	97.3%	0.00091
17	60	30	60	96.2%	96.8%	0.0011
18	90	−25	50	97.2%	98.3%	0.00032
19	90	−20	5	98.2%	98.3%	0.00016
20	90	−10	10	98.2%	98.4%	0.00020
21	90	10	20	98.2%	98.4%	0.00028
22	90	20	25	98.4%	98.4%	0.00033
23	90	25	50	97.9%	98.1%	0.00045
24	90	30	60	97.8%	98.2%	0.00051
25	120	0	25	98.9%	98.8%	0.00017
26	120	−25	50	98.3%	98.9%	0.00019
27	120	−20	5	98.4%	98.7%	0.00011
28	120	−10	10	98.7%	98.8%	0.00013
29	120	10	20	98.8%	98.7%	0.00017
30	120	20	25	98.8%	98.8%	0.00020
31	120	25	50	98.8%	98.8%	0.00025
32	120	30	60	98.6%	98.8%	0.00028
33	150	−25	50	98.7%	99.0%	0.00012
34	150	0	50	98.8%	99.1%	0.00014
35	150	−20	5	98.8%	98.8%	0.000079
36	150	−10	10	98.8%	98.9%	0.000089
37	150	10	20	99.0%	98.8%	0.00011
38	150	20	25	98.9%	98.8%	0.00012
39	150	25	50	99.0%	99.1%	0.00016
40	150	30	60	98.8%	99.1%	0.00017
41	90	10	10	66.3%	65.3%	0.00018
42	90	30	30	66.3%	65.6%	0.00028
43	90	30	30.1	76.8%	78.2%	0.00032
44	150	10	10	67.2%	66.8%	0.000093
45	150	30	30	67.1%	66.6%	0.00012
46	150	30	30.1	77.2%	79.1%	0.00013

The first column gives the row number. The next three columns gives the values of the pumping strength Ω, and the relative detunings *δ*_*a*_ − *δ*_*σ*_, *δ*_*b*_ − *δ*_*σ*_. For each row, the value of *δ*_*σ*_ is chosen such that *p*_*ab*_ is at its maximum in steady-state analysis as shown in Fig. 2a. The fifth column gives the fidelity for heralding a single photon state in mode *b* conditioning on detecting a photon in mode *a*, and vice versa in the sixth column. The last column gives the rate for producing photon pairs with one photon in each mode. Values for other parameters are *g*_*a*_ = *g*_*b*_ = 1, *κ*_*a*_ = *κ*_*b*_ = 0.1, *γ*_*σ*_ = 0.01. We have normalized the cavity-emitter coupling constant to be unity. All other coupling strengths, frequency detunings and decay rates are in units of them. In experiments in the optical regime, for example, the cavity-emitter coupling constants would be in the megahertz regime^15–17^.  In row number 1–40 the pumping strength increases gradually. Since the computations are very time consuming, the various parameters are sampled in a Monte-Carlo-like way. In row number 41–46, the resonant frequencies of the two cavities are tuned very close together to showcase that this situation is to be avoided as discussed in the text. For more details please see the text.

**Table 3:** Dependence of the heralding fidelity on the decay rates.

Row no.	Ω	(*δ*_*a*_ − *δ*_*σ*_)	(*δ*_*b*_ − *δ*_*σ*_)	Fidelity (*a* detected)	Fidelity (*b* detected)	Rate	*κ*_*a*_,*κ*_*b*_	*γ*_*σ*_
1	150	25	50	99.0%	99.1%	0.00016	0.1	0.01
2	150	25	50	94.6%	94.7%	0.000062	0.3	0.01
3	150	25	50	98.5%	98.5%	0.00016	0.1	0.03
4	150	25	50	97.3%	97.5%	0.00011	0.12	0.077
5	10	−25	50	59.3%	88.3%	0.00045	0.1	0.01
6	10	−25	50	21.2%	64.8%	0.00019	0.3	0.01
7	10	−25	50	56.0%	87.8%	0.00042	0.1	0.03
8	10	−25	50	39.3%	83.3%	0.00029	0.12	0.077
9	20	−10	10	79.9%	91.2%	0.00053	0.1	0.01
10	20	−10	10	60.3%	85.2%	0.00050	0.3	0.01
11	20	−10	10	84.8%	89.4%	0.0012	0.1	0.03
12	20	−10	10	83.8%	86.5%	0.0019	0.12	0.077
13	60	10	20	97.4%	97.6%	0.00050	0.1	0.01
14	60	10	20	92.5%	93.5%	0.00029	0.3	0.01
15	60	10	20	96.0%	96.0%	0.00071	0.1	0.03
16	60	10	20	95.0%	95.2%	0.00062	0.12	0.077

The meaning of the first seven columns are the same as in Table 2. The last two columns are the values of the cavity decay rate and the spontaneous emission rate of the emitter, respectively. We have assumed *κ*_*a*_ = *κ*_*b*_. Values for other parameters are *g*_*a*_ = *g*_*b*_ = 1. We have normalized the cavity-emitter coupling constant to be unity. For more details please see the text.

**Table 4:** Dependence of the heralding fidelity on the coupling constants.

Row no.	Ω	(*δ*_*a*_ − *δ*_*σ*_)	(*δ*_*b*_ − *δ*_*σ*_)	Fidelity (*a* detected)	Fidelity (*b* detected)	Rate	*κ*_*a*_,*κ*_*b*_	*γ*_*σ*_	*g*_*a*_	*g*_*b*_
1	60	10	20	97.4%	97.6%	0.00050	0.1	0.01	1	1
2	60	10	20	99.1%	92.6%	0.000095	0.1	0.01	0.3	1
3	60	10	20	92.0%	99.1%	0.000096	0.1	0.01	1	0.3
4	60	10	20	95.0%	95.2%	0.00062	0.12	0.077	1	1
5	60	10	20	97.3%	79.8%	0.000064	0.12	0.077	0.3	1
6	60	10	20	79.1%	97.5%	0.000065	0.12	0.077	1	0.3

The meaning of the first 9 columns are the same as in Table 3. The last two columns are the values of the coupling constants of the emitter to cavity mode *a* and *b* respectively. We have assumed *κ*_*a*_ = *κ*_*b*_. We have normalized the larger cavity-emitter coupling constant to be unity. For more details please see the text.

In Table 3 we investigate the influence of larger decay rates. As can be seen, in general increasing the decay rates will decrease the heralding fidelity. Nevertheless, even if the cavity decay rate is 3 times higher as compared to that in Table 2, a fidelity of 94.7% can still be attained, as can be seen from row number 2 of Table 3. If the spontaneous emission rate of the emitter is 3 times higher than in Table 2, a fidelity as high as 98.5% can nonetheless be achieved, as shown in row number 3 of Table 3.

An interesting dependence of the heralding fidelity on the cavity-emitter coupling constants are shown in Table 4. As can be seen by comparing, e.g., row 3 with row 1, quite counterintuitively, starting from equal couplings of the emitter to both modes, decreasing the coupling to one of the modes can lead to an increase in heralding fidelity from 97.6% to 99.1%.

## Discussion

In the present work, we have described a protocol for heralding single photon states with near-unity fidelity using the two-mode Jaynes Cummings model. This protocol can be realized in existing state-of-the-art cavity quantum electrodynamical system consisting of neutral atoms and Fabry-Perot type cavities^33^. In such systems, one can have *κ*/*g* = 0.12, *γ*_*σ*_/*g* = 0.077, where *κ* is the cavity decay rate while *g* is the coupling strength between the cavity and the two-level atom, and *γ*_*σ*_ is the spontaneous emission rate of the atom. As shown in row 4 of Table 4, under such parameters a fidelity of 95.2% can be achieved. By using a *smaller* coupling constant to one of the modes, the fidelity can further increase to 97.5%, as seen in row number 6 of Table 4.

In ref.^29^, it was firstly proposed that the strongly pumped Jaynes-Cummings model can be used to produce high-purity photon pairs. However, since the two photons thus produced are identical, it is not easy to separate them cleanly. As a result, it is not easy to use them to herald single photons with near-unity fidelity. In the present work, by employing two cavity modes with different energies, we show that one can produce high-purity photon pairs with each photon in a different mode. These two photons *can* be separated cleanly, and thus can be used to herald single photons with high fidelity.

The two-mode cavity employed in our proposal can probably be readily realized using Fabry Perot type cavities as that described in ref.^33^. In such cavities, two modes with orthogonal polarizations are natrually present. Also, due to birefringence, their resonant frequencies are different, just as required by our protocol.

At present, our new protocol is perhaps not yet more advantageous than mature protocols^10,15–17^ using cavity quantum electrodynamical systems in the strong coupling regime that generate single photon deterministically, or heralded single photon sources using spontaneous parametric down conversion^34–37^, which is very convenient experimentally. Still, our work shows clearly the usefulness of employing the multiphoton resonance between the laser dressed states, and that the paradigmatic Jaynes-Cummings model can be used to produce high purity separable photon pairs. This is an important first step toward more refined protocols. For example, if the effective coupling strength from the zero-photon manifold to the two-photon manifold can be somehow enhanced, the pair production rate can be further increased. Or, if the spontaneous emission rate can somehow be suppressed, our protocol will actually produce photon pairs on demand^28^. Also it is readily conceivable that through extending our work by employing more than one atom, or by working at higher order resonance than the two-photon resonance here, it is probable that one can generate with high purity three photon or even four photon states, in a way such that one can herald with high fidelity two photon or three photon states. These are still very challenging tasks for existing protocols. Finally, to our best knowledge the physical processes involved in the present proposal, while already realizable, has not been tested experimentally in the optical regime and so is very interesting physically in its own right.

It will be interesting to investigate more deeply why the counterintuitive dependence on coupling constant happens: a decrease in the coupling of the emitter to one of the modes can lead to an increase in heralding fidelity, cf. Table 4 and the last part of Results.

In practical implementation, it is possible that the coupling constants of the emitter to one of the two modes is less than optimal. Nevertheless, this may not necessarily reduce the heralding fidelity. Indeed, as discussed above, the heralding fidelity may actually increase.

Usually, increasing the cavity-emitter coupling constants is a very painstaking task experimentally. It thus merits further investigation whether the above phenomenon has wider implications. Namely, probably the same underlying mechanism is also at work or can be engineered to work in some other application scenarios. If so, a lower coupling may actually be better and one no longer need and in fact preferably not painstakingly increase the coupling constant.

## Methods

The steady-state density matrix *ρ*_*ss*_ is calculated by requiring $\frac{d{\rho }_{ss}}{dt}=0$. The quantities shown in Fig. 2a, such as *p*_*a*_, *p*_*ab*_ are calculated using *ρ*_*ss*_. For example, ${p}_{a}={\rm{Tr}}[|a\rangle \langle a|{\rho }_{ss}]$, ${p}_{ab}={\rm{Tr}}[|ab\rangle \langle ab|{\rho }_{ss}]$, where Tr[] means trace, while |a> is the Fock state with one photon in mode *a* satisfying *a*^†^*a* |*a*〉 = |*a*〉 and *b* |*a*〉 = 0, and |*ab*〉 is the Fock state with one photon in each mode.

The quantum trajectory simulation are performed in the following way: starting with a normalized initial state |*ϕ*_0_〉, pick randomly a number *r* between 0 and 1. The state is then evolved according to

4 $$i\frac{\partial \varphi }{\partial t}={H}_{{\rm{eff}}}\varphi ,$$

where

5 $${H}_{{\rm{eff}}}=H-\frac{i}{2}{\kappa }_{a}{a}^{\dagger }a-\frac{i}{2}{\kappa }_{b}{b}^{\dagger }b-\frac{i}{2}{\gamma }_{\sigma }{\sigma }_{+}{\sigma }_{-}.$$

At the time when 〈*ϕ*|*ϕ*〉 decreases to *r*, perform a quantum jump on the state: Either a cavity decay in mode *a* or mode *b*, or a spontaneous emission of the emitter is performed and recorded. Correspondingly the state |*ϕ*〉 is changed into $\frac{a|\varphi \rangle }{|a|\varphi \rangle |}$, $\frac{b|\varphi \rangle }{|b|\varphi \rangle |}$, or $\frac{{\sigma }_{-}|\varphi \rangle }{|{\sigma }_{-}|\varphi \rangle |}$ where || means taking the norm. Which jump to perform is determined by a random pick subject to the relative probability: *p*(*a*|*ϕ*〉) : *p*(*b*|*ϕ*〉) : *p*(*σ*_*−*_|*ϕ*〉) = *κ*_*a*_〈*ϕ*|*a*^†^*a*|*ϕ*〉 : *κ*_*b*_〈*ϕ*|*b*^†^*b*|*ϕ*〉 : *γ*_*σ*_〈*ϕ*|*σ*_+_*σ*_−_|*ϕ*〉. Then the state is normalized and the above procedure is repeated.
